# Role of Heterogeneous Nuclear Ribonucleoproteins in the Cancer-Immune Landscape

**DOI:** 10.3390/ijms24065086

**Published:** 2023-03-07

**Authors:** Meenakshi Sudhakaran, Andrea I. Doseff

**Affiliations:** 1 Molecular, Cellular, and Integrative Physiology Graduate Program, Michigan State University, East Lansing, MI 48824, USA; 2 Department of Physiology, Michigan State University, East Lansing, MI 48824, USA; 3 Department of Pharmacology and Toxicology, Michigan State University, East Lansing, MI 48824, USA

**Keywords:** macrophages, myeloid, immune regulation, apoptosis, cancer resistance

## Abstract

Cancer remains the second leading cause of death, accounting for approximately 20% of all fatalities. Evolving cancer cells and a dysregulated immune system create complex tumor environments that fuel tumor growth, metastasis, and resistance. Over the past decades, significant progress in deciphering cancer cell behavior and recognizing the immune system as a hallmark of tumorigenesis has been achieved. However, the underlying mechanisms controlling the evolving cancer-immune landscape remain mostly unexplored. Heterogeneous nuclear ribonuclear proteins (hnRNP), a highly conserved family of RNA-binding proteins, have vital roles in critical cellular processes, including transcription, post-transcriptional modifications, and translation. Dysregulation of hnRNP is a critical contributor to cancer development and resistance. HnRNP contribute to the diversity of tumor and immune-associated aberrant proteomes by controlling alternative splicing and translation. They can also promote cancer-associated gene expression by regulating transcription factors, binding to DNA directly, or promoting chromatin remodeling. HnRNP are emerging as newly recognized mRNA readers. Here, we review the roles of hnRNP as regulators of the cancer-immune landscape. Dissecting the molecular functions of hnRNP will provide a better understanding of cancer-immune biology and will impact the development of new approaches to control and treat cancer.

## 1. Introduction

Cancer is the second leading cause of mortality worldwide, accounting for approximately 10 million deaths in 2020 [1]. The prevalence of cancer has increased in the last decade, with a significant burden disproportionately inflicted on socioeconomically underprivileged and racially underrepresented populations [2]. Tumors constitute complex and heterogeneous ecosystems where cancer cells co-exist in the tumor microenvironment (TME) with myeloid cells, among other cell types. Cancer cells bear genomic and proteomic alterations that control their fate [3]. Myeloid cells, including monocytes, macrophages, and myeloid-derived suppressor cells (MDSC), play a critical role in tumor progression by fueling cancer cell growth, increasing metastasis, and promoting resistance to therapy [4,5]. The increased number of myeloid cells found in the TME is associated with poor clinical prognosis [6]. Consistently with these observations, chemical or genetic ablation of macrophages resulted in delayed tumor growth and an improved response to therapy [7]. The dynamic crosstalk in the TME between cancer and immune cells defines the cancer immune landscape, which is a decisive factor in the evolution of tumors and their response to therapy [3]. Emerging findings during the last few years highlight the relevance of this crosstalk, but how it is regulated remains poorly understood.

Cancer-specific proteome aberrations, affecting 60% of the total proteome, promote uncontrolled cell proliferation, resistance to cell death, and the production of recruiting factors that attract myeloid cells into the TME. The heterogeneous ribonuclear proteins (hnRNP) are essential architects of proteome diversity. The hnRNP comprise a highly evolutionary conserved family of RNA-binding proteins (RBP) involved in basic cellular mechanisms, including post-transcriptional regulation (e.g., splicing), mRNA stability, and protein translation, all of which are critical contributors to proteome complexity [8]. Cancer cells exhibit a higher expression of hnRNP than non-cancer cells [9,10,11,12]. HnRNP function as tumor drivers by promoting cancer cell proliferation, invasion, inflammation, resistance to cell death, deregulation of metabolic homeostasis, and therapeutic resistance [13]. These effects are mainly mediated by the ability of the hnRNP to bind RNA-specific sequences, thereby enhancing the stability of mRNA or promoting alternative splicing (AS) isoforms that promote cancer growth and resistance to anti-cancer treatments [14,15,16]. Recent studies showed that hnRNP can also associate with transcription factors, acting as co-transcriptional regulators. They can also modify the chromatin structure or promote epigenetic alterations [17,18,19]. While their contribution to cancer cell growth and therapeutic resistance is well-accepted, their role in immune cell function has been less studied. HnRNP modulate immune responses of both innate and adaptive immune cells by regulating expression or AS [20,21,22,23]. For example, hnRNPA0 enhances mRNA stability by binding to specific sequences found in the mRNA of inflammatory genes such as tumor necrosis factor (TNF)-α and interleukin (IL)-6 [23]. This effect results in the increase of inflammatory cytokines, which are critical modulators of cancer-immune crosstalk. Here, we present an integrated view of hnRNP in cancer, including their emerging roles in myeloid immune cells. We address unmet needs to increase our understanding of how hnRNP mechanistically impact the cancer-immune landscape and propose potential roles of hnRNP in modulating the crosstalk between tumor and myeloid cells, which may help unveil new targets for the prevention and treatment of cancer.

## 2. Structure and Regulation of HnRNP

**Figure 1:**
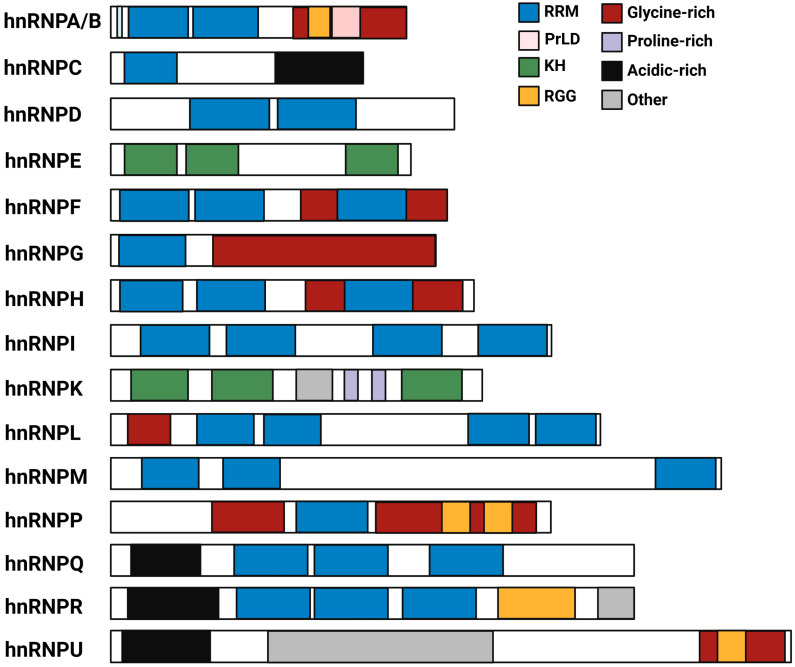
Models representing the most conserved domains found in the mammalian hnRNP protein family. RNA recognition motif (RRM), prion-like domain (PrLD), K homology (KH), and arginine–glycine–glycine repeat (RGG).

The hnRNP were first identified as proteins associated with heterogeneous RNA in monkey kidney fibroblast cell line CV-1 and isolated as part of the 40S mRNA-protein complex by sucrose density gradients [24,25]. The hnRNP family comprises 33 proteins characterized by the presence of one or more RNA-binding domains (RBD) containing amino acid consensus sequences that mediate their interaction with RNA [26]. There are three different types of RBD, including the RNA recognition motif (RRM), the K homology (KH) domain, and the arginine–glycine–glycine repeat (RGG) box (Figure 1). RRM is the most common RBD, found in more than 80% of all the hnRNP, and characterized by the presence of four β-sheets and two α-helices (βαββαβ). RRM domains contain the highly conserved octamer RNP1 and hexamer RNP2 motifs crucial for their binding to RNA. The variable loops connecting the β-sheets contribute to the RNA-binding specificity of hnRNP [27]. The KH domain, found originally in hnRNPK, is characterized by the presence of a three-stranded anti-parallel β-sheet packed against three α-sheets (βααββα). The two α-helices are linked through an extended loop enabling interaction with RNA [28]. HnRNP such as hnRNPA1, hnRNPA2/B1, hnRNPP, hnRNPR, and hnRNPU have RGG boxes in their carboxy-terminal domains characterized by adjoining sequences containing aromatic phenylalanine and tyrosine residues interspersed between arginine and glycine amino acids [29]. Despite the high similarity in the RBD, how hnRNP define their unique RNA-binding specificity remains inadequately understood.

The hnRNP also have other auxiliary domains, consisting of proline-, glycine-, or acidic-rich domains. The glycine-rich domains (GRD), mostly found in the carboxy-terminal, are often responsible for interactions with proteins, including other hnRNP (Figure 1) [26]. The GRD of hnRNPA1 and hnRNPA2/B1 contains a distinctive prion-like domain (PrLD), enriched in charged polar amino acids and glycine, which is essential for the assembly of RBP granules [30].

**Figure 2:**
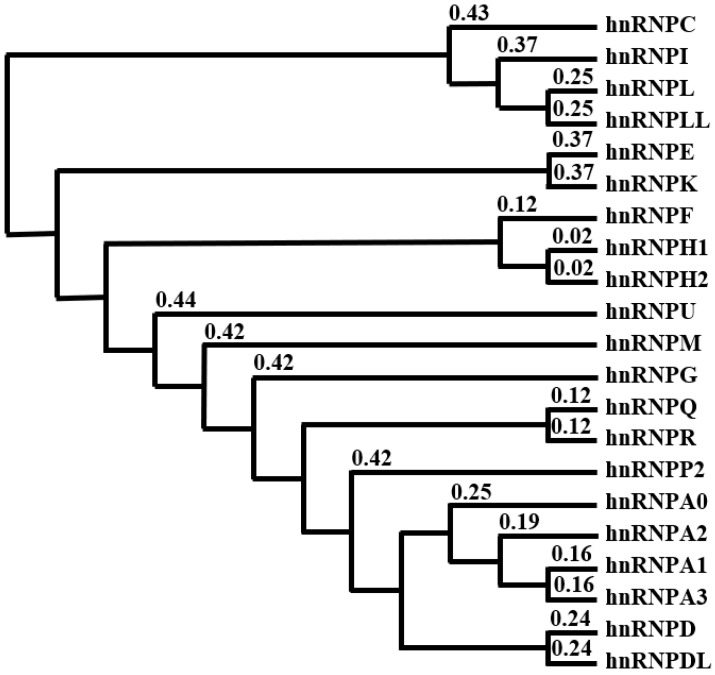
Phylogenetic tree of hnRNP based on human amino acid sequences generated using ClustalW2-Phologeny (ClustalW2 Phylogenetic Tree < Phylogeny < EMBL-EBI) [48]. The numbers above each node indicate the phylogenetic confidence of the tree topology.

Based on the amino acid sequence, the primary hnRNP homology resides in their RBD. For instance, the hnRNPA/B subfamily, comprising hnRNPA0, hnRNPA1, hnRNPA2/B1, and hnRNPA3, shares high amino acid homology (Figure 2). HnRNPA1 and hnRNPA3 are the closest members, with 90% homology. Similarly, hnRNPA2/B1 and hnRNPA1 share approximately 80% amino acid homology at their RRM, but only 50% similarity in the carboxy-terminal GRD regions [14]. The hnRNPA/B subfamily possesses the slightest resemblance with hnRNPC, hnRNPI, and hnRNPL, as depicted in the phylogenetic tree (Figure 2). HnRNP are evolutionarily conserved. Several hnRNP found in *Drosophila melanogaster* show 60% homology to mammalian hnRNP. For instance, the proteins encoded by the *Drosophila Hrb98DE* and *Hrb32AB* loci are similar in sequence to the mammalian hnRNPA2/B1 [31]. Hrp59, found in the midges *Chironomus tentans*, is structurally similar to hnRNPM with three RRM domains [32]. These findings suggest that hnRNP are structurally conserved between vertebrates and invertebrates.

HnRNP are differentially distributed in the cell and their functions are controlled in part by their localization. Most hnRNP contain nuclear localization signals responsible for their nuclear-cytoplasmic shuttling [33]. Despite the similarity in domains, hnRNPA/B subfamily proteins hnRNPA1, hnRNPA2/B1, and hnRNPA3 are differentially localized in the cell. HnRNPA1 resides around the nuclear envelope, while hnRNPA2/B1 and hnRNPA3 are concentrated in the nucleoplasm, as demonstrated using immunostaining [34]. All of them are also found in the cytoplasm [35,36,37]. Treatment with RNase A decreased the majority of hnRNPA/B proteins in the nucleus compared to DNase I, suggesting that their nuclear localization depends on the association with RNA [34]. Additionally, hnRNP are regulated by post-translational modifications, including phosphorylation, ubiquitylation, sumoylation, and methylation, which control their cellular localization, interaction with other proteins, and biological functions [38,39,40,41,42,43,44].

Most hnRNP exist in several different isoforms due to AS [8]. HnRNPA2/B1 pre-mRNA undergoes AS to generate four different isoforms, wherein B1 and A2 are the most predominant, with the latter lacking exon 2. Despite their sequence similarity, cross-linking immunoprecipitation (eCLIP) experiments identified some differences in the binding preferences between hnRNPA2 and hnRNPB1 in the human breast cancer cell line MCF-7. While most bound transcripts were common, around 30% were unique to hnRNPA2 or hnRNPB1 [45], demonstrating that they have a set of non-overlapping cellular targets. In some cases, the isoforms can regulate their own expression. For example, hnRNPD and hnRNPDL control their expression through AS, which introduces exon 8 in the latter’s 3′ untranslated region (UTR) to promote nonsense-mediated decay (NMD) [46]. HnRNPA1 and hnRNPA2/B1 modulate their expression in a compensatory manner at both RNA and protein levels, an effect that is mediated through the 3′UTR regions, as demonstrated by luciferase reporter and reverse-transcriptase polymerase chain reaction assays [47].

The expression of hnRNP and their AS isoforms vary in distinct tissues and physiological conditions [49,50]. The level of abundance of different AS isoforms may affect their binding preference for mRNA. For example, hnRNPA2 binds to mature transcripts comprising more exonic and UTR regions than hnRNPB1, which was attributed to the higher abundance of the former isoform in breast cancer cell lines, as suggested by eCLIP experiments [45]. Additionally, the expression levels of hnRNP vary throughout different stages of the cell cycle. HnRNPA2 protein levels were enriched during G1 and significantly decreased in S and G2/M phases in the human HeLa cell line [34,51]. Increased levels of hnRNP are observed in several disease conditions, including cancer, autoimmune, and neurodegenerative diseases [8]. Few studies reported aberrant transcription of hnRNP in disease conditions. Depletion of the transcription factor c-Myc reduced the expression of hnRNPU, hnRNPI, hnRNPA1, and hnRNPA2/B1 in human hepatocellular carcinoma and mouse fibroblasts, suggesting that c-Myc oncogene controls their expression [52,53]. A recent study showed that the transcription factor octamer-binding transcription factor 4 (OCT4) regulates the expression of hnRNPA2/B1 in porcine trophectoderm cell lines using luciferase reporter assays [54]. Additionally, the nuclear factor kappa-light-chain-enhancer of activated B cells (NF-κB)-RelA (p65 subunit) and specificity protein (Sp)-1 bind to the hnRNPD and hnRNPK promoters, respectively, as shown by chromatin immunoprecipitation (ChIP) and electrophoresis mobility shift assays (EMSA) in oral and breast cancer cells [55,56]. However, it remains unclear how the expression of hnRNP at the transcriptional level is regulated in non-cancer and immune cells. Further studies are called for to understand the gene regulatory networks controlling hnRNP transcription in normal and disease conditions.

## 3. Functions of hnRNP

As a core component of the RBP splicing complex, hnRNP have essential cellular roles during post-transcriptional regulation. The major functions of hnRNP in splicing and mRNA stability are well-known, but their emerging roles as co-transcriptional regulators and mRNA readers call for better understanding. Deciphering the functions of hnRNP in normal and abnormal conditions will be impactful in designing new approaches to prevent and treat diseases. Here, we summarize some of the significant known biological functions of hnRNP and those recently emerging.

### 3.1. DNA Maintenance

Genomic instability, a hallmark of cancer, promotes aberrant cell survival [3,57]. The cells adapt to prevent defects due to genome instability by initiating DNA damage responses [58]. HnRNP play a pivotal role in sensitizing the response to DNA damage by binding to double-stranded DNA breaks (DSB). For instance, hnRNPC1 and its AS isoform hnRNPC2 can directly bind to gamma radiation-induced DSB and promote DNA repair in HeLa cells [59]. Similarly, hnRNPUL1 is recruited to DSB and co-localized with phosphorylated histone H2AX, a marker of DNA damage. HnRNPUL1 is recruited to DSB and reduces the sensitivity to DNA damage by associating with poly (ADP-ribose) polymerase 1 (PARP1), a key enzyme in DNA repair [60]. HnRNPR binds to Y-box binding 1 (Yb1) protein, which is recruited upon DNA damage to interact with phosphorylated H2AX at DSB [61]. HnRNP also regulate the DNA damage response by affecting the ataxia telangiectasia mutated (ATM) or ATM-rad3-related (ATR)-dependent signaling pathways. HnRNPUL1 is recruited to the DNA damage site and associates with DSB sensor complex MRE11-RAD50-NBS1. Depletion of hnRNPUL1 suppressed the ATR signaling pathway and inhibited the nucleolytic processing of DNA ends required for DSB repair by homologous recombination [62]. ATM phosphorylated HnRNPK, which enhanced its stability and function as a p53 cofactor to induce DNA damage response gene expression [63]. However, HnRNPB1 functions as a negative regulator of DNA repair. HnRNPB1 associates with DNA-protein kinase (DNA-PK), a serine/threonine protein kinase involved in non-homologous end-joining DNA repair, upon irradiation, inhibiting its enzyme activity. HnRNPB1 knockdown, using siRNA, restored DNA-PK kinase activity and DNA repair in gamma-irradiated human lung cancer cells [64,65]. Additionally, DNA-PK phosphorylates several hnRNP, including hnRNPC1 and hnRNPU [66,67]. However, the functional impact of this phosphorylation is not clear.

In addition to its role in DNA repair, hnRNP also regulate normal DNA homeostasis by maintaining telomere stability. Telomeres are crucial in maintaining chromosome integrity and genomic stability in eukaryotic cells. Telomere length, maintained by the telomerase enzyme, acts as a mitotic checkpoint, triggering either cellular senescence or uncontrolled proliferation [68]. HnRNPA2/B1 associates with human telomerase RNA component (hTERC), thereby regulating its activity and promoting telomere elongation, as demonstrated using in vitro and in vivo studies [69,70]. A splice variant of hnRNPA2/B1, called hnRNPA2*, lacking exons 7, 8, and 9 in the GRD, also binds to telomeric DNA, suggesting that RRM is required for telomeric binding [71]. However, on the contrary, recent studies showed that hnRNPA2 reduces telomere activity through acetylation at histone H4K8 in mitochondrial dysfunctional cells. Mutations on the RRM domain of hnRNPA2 containing lysine acetyltransferase (KAT) activity enhanced telomerase activity, resulting in an increased telomere length due to impaired H4K8 acetylation [72]. The RRM domains of hnRNPF, hnRNPH, and hnRNPD also interact with hTERC, contributing to an increase in the telomere length [73,74,75]. HnRNPA1 or hnRNPA2/B1 deficiency altered the distribution of telomere 3′ G-rich overhangs, G-tail, and lengths [70]. However, a different study using hnRNPA1 and its GRD-lacking isoform UP1 showed that only the latter was able to restore telomerase activity in hnRNPA1-deficient cells, while both isoforms can bind to single-stranded telomeric repeats [76]. These somehow contradictory observations on the role of hnRNPA1 in regulating telomerase activity might be due to the use of different cell types for the studies and hence require further validation. HnRNPD binds to human telomeric repeat through its C-terminal domain and maintains telomere G-tails through the destabilization of quadruplex DNA structure for the elongation by telomerase [77]. Overall, these studies highlight the role of hnRNP in maintaining genomic stability by promoting telomerase activity, telomere elongation, and the DNA damage response, suggesting a potential mechanism by which they promote uncontrolled proliferation in cancer cells [78].

### 3.2. Transcriptional Regulation

Recent studies revealed that hnRNP can regulate transcription by either functioning as a transcriptional cofactor or a chromatin remodeler. HnRNP can control transcription by directly associating with either transcription factors or DNA at the promoter regions. For example, hnRNPA2 seems to bind at the *COX-2* promoter, a gene involved in tumor progression, and upregulates the production of inflammatory cytokine PGE_2_ in lung cancer cell lines [79]. These results propose that hnRNPA2 could directly bind to DNA, but how this association confers increased *COX-2* transcription remains unknown since hnRNPA2 lacks transcriptional activity on its own. HnRNPA2 also promotes transcription by associating with the enhanceosome to increase the occupancy of transcription factors, including c-Rel, CREB, and C/EBPδ, in the promoter of the mitochondrial stress genes *Cathepsin L* and *Glut-4*, as shown by reporter gene and ChIP assays in mitochondrial DNA-depleted murine myoblasts. Mechanistic studies using hnRNPA2 RRM and GRD show that the former is critical for the regulation of transcription as it binds to the *Cathepsin L* and *Glut-4* promoters [80]. Mutations of arginine 48 and 50 in hnRNPA2, located in its acetyltransferase activity domain, impaired *Cathepsin L* and *Glut-4* transcription [77]. These studies suggest that hnRNPA2 KAT activity may contribute to its ability to regulate transcription. To date, amongst all the hnRNP family proteins, only hnRNPA2 showed KAT activity. This finding calls for additional studies to explore whether other hnRNP family members possess this function. Additionally, further investigation is needed to understand whether the transcriptional activity of hnRNPA2 necessitates direct interaction with DNA at the promoter, citing an unmet need for additional investigation.

Several studies showed that hnRNP associate with specific DNA sequences in promoters, inducing chromatin structural changes and hence leading to gene expression alterations. HnRNP associate with and unfold polypurine-polypyrimidine-rich G-quadruplexes (G4) and cytosine-rich iMotifs, found in the promoters to induce transcription [81]. HnRNPA1 increased *TRA2B* and *KRAS* transcription by interacting with G4 regions in their promoters [82,83]. HnRNPK binds to cytosine-thymine (CT) tracts found in the *c-Myc* promoter to enhance its expression [84]. The recruitment of hnRNPK to the CT regions found in the *egr-1* promoter upregulates its expression, resulting in increased proliferation of human colon carcinoma cell lines [85]. Mutations in the CT tracts reduced the association of hnRNPK to DNA, leading to the inhibition of transcriptional activity [84,85]. HnRNPLL binds to the i-Motif found on the promoter of *BCL2*, an apoptotic inhibitor, as demonstrated by EMSA and surface plasmon resonance analyses. This resulted in chromatin unfolding, leading to increased transcription and consequent resistance to cell death [86]. Gene expression is also regulated through RNA activation (RNAa), wherein small activating dsRNA (saRNA) binds complementary to genomic sequences around the promoter region of genes to enhance the transcriptional output. HnRNPA1 and hnRNPA2/B1 interact with saRNA and are required for RNAa to induce transcriptional activation of tumor suppressor gene *p21*, as shown using in vitro and in vivo studies [87].

Several hnRNP associate with transcription cofactors acting as transcriptional co-activators. HnRNPD can associate with the TATA-binding protein (TBP), KAT p300, and DNA [88]. HnRNPK also binds to TBP, enhancing transcription, as demonstrated by reporter gene and immunoprecipitation assays [89]. Post-translational modifications such as phosphorylation seem to enhance hnRNP transcriptional cofactor activity. For example, phosphorylation of hnRNPA2 at serine 219 and threonine 98 by Protein kinase B (Akt) enhanced its KAT activity, resulting in increased transcriptional activity [17,39,80]. Similarly, the transcriptional activity of hnRNPD is regulated through phosphorylation at serine 87 by Protein Kinase A (PKA) [88].

Few studies showed that hnRNP can also act as transcriptional repressors. HnRNPK suppresses the transcription of osteocalcin, a gene involved in energy metabolism, by removing transactivators from CT tracts found in the promoter region [90]. HnRNPK, along with DNA-binding protein Pur-α, binds to single-stranded DNA structures found in the *CD43* promoter to inhibit transcription [91]. Overall, these studies emphasize the role of hnRNP as transcriptional regulators. Most studies of hnRNP as transcriptional regulators were performed using cancer cell lines. Hence, additional studies would be helpful to reveal the role of hnRNP in transcriptional regulation in non-cancer and immune cells.

### 3.3. Alternative Splicing

Alternative splicing is a major contributor to proteomic complexity by promoting the expression of different mRNA arising from the same gene [92]. RBP are key regulators of AS. Some hnRNP are part of the spliceosome, binding to the exon or intron splicing silencers (ESS and ISS) and often antagonizing the effect of serine/arginine (SR) proteins [93]. A novel understanding of splicing patterns generated by individual hnRNP is emerging due to the advent of genome-wide methods for detecting the direct binding sites of RBP on their RNA substrates, coupled with technologies such as RNAseq. The sites of interactions of six hnRNP, hnRNPA1, hnRNPA2/B1, hnRNPU, hnRNPH, hnRNPM, and hnRNPF, with ~10,000 pre-mRNAs, were identified using CLIP-seq analyses to reveal RNA splicing maps [94]. HnRNP bind in a position-specific pattern around cassette exons, wherein an intervening exon between two other exons is either included or excluded, to regulate AS in human 293T cells. While hnRNP-binding sites are primarily intronic, enrichment of hnRNPA1, hnRNPA2/B1, hnRNPU, and hnRNPF was seen within exonic and 3′UTR regions. Interestingly, 71% of the cancer-associated genes found in the Sanger-COSMIC database (COSMIC: Catalogue of Somatic Mutations in Cancer (sanger.ac.uk)) are direct targets of at least one of the six hnRNP [94,95].

HnRNPA1 and hnRNPA2/B1 inhibit 5′ and 3′ splicing site recognition and promote distal 5′ splicing site selection [96]. HnRNA1 and hnRNPA2 alter the activity of the metabolic enzyme Pyruvate Kinase (PKM) through exon 10 inclusion to increase the PKM2 isoform, thereby increasing the ratio of PKM2/PKM1 isoforms in cancer cells. An increase in PKM2 shifts the glucose metabolism to aerobic glycolysis in cancer cells, which has profound implications in promoting uncontrolled cancer growth by inducing metabolic rewiring to glycolysis-dependent cell proliferation [97]. HnRNPK and nuclear speckle-associated protein bind influenza virus M-mRNA downstream of the M2 5′ splice site, leading to AS, which enhances viral replication [98]. HnRNPG prevents the inclusion of the exon between splicing sites SA3358 and SD3632 on the human papillomavirus (HPV)-16 late L1 mRNAs by binding to the 8-nucleotide regulatory RNA enhancer element [99]. Genome-wide analyses suggest that hnRNPK regulates AS of transcription factors, including STAT6 and IRF1, involved in immune responses [100]. HnRNPM retained intron 3 of *IL-6* pre-mRNA, which served as a rate-limiting step in the processing of *IL-6* mRNA, thereby halting IL-6 production [21]. HnRNP can also promote exon/intron exclusion. The siRNA-mediated knockdown of hnRNPA2/B1 excludes the exon 2 located at the *TP53INP2* 5′UTR [101]. Similarly, hnRNPD binds to HPV16 mRNA to execute intron retention and the consequent production of E1 and E6 proteins, which are key for viral DNA replication [102]. How hnRNP can promote both inclusion and exclusion of exons/introns is not fully understood.

Several hnRNP can splice themselves. For example, hnRNPA2/B1 regulates the abundance of its mRNA by AS of the 3′UTR to promote the production of isoforms degraded by nonsense-mediated mRNA decay [103]. Similarly, hnRNPD and hnRNPDL control their expression by AS of cassette exons in their 3′UTRs [46]. Overall, hnRNP are key players in programming AS circuitry towards aberrant cancer transcriptomes. Nevertheless, how changes in AS in cancer-specific genes affect the functional domains of their proteins is not fully understood. This knowledge would be invaluable in identifying potentially targetable proteins to control cancer. Studies delineating the functional impact of hnRNP-driven AS in proteome are warranted.

### 3.4. mRNA Readers

*N*^6^-methyladenosine (m^6^A) is a modification wherein methyl groups are added to the N6-position of adenosine in eukaryotic mRNAs and noncoding RNAs. This post-transcriptional modification is emerging as a regulator of splicing, mRNA stability, and translation. M^6^A predominantly occurs in stop codons, 3′UTRs, and long exons. M^6^A modifications are mediated by methyltransferases (a.k.a writers), which add methyl groups, removed by demethylases (a.k.a erasers), and identified by m^6^A-binding proteins (a.k.a readers) [104]. Dysregulation of m^6^A modification contributes to tumor initiation, progression, and response to anti-cancer drugs [105]. HnRNP have been recently recognized as m^6^A readers. M^6^A facilitates the binding of hnRNPK to a UUUUU-tract in mRNA and long non-coding RNA (lncRNA) by promoting RNA unfolding [106]. The function of hnRNPA2/B1 as an m^6^A reader remains controversial. The initial findings using high-throughput sequencing coupled with CLIP (HITS-CLIP) showed that hnRNPA2/B1 binds to m^6^A-bearing sites in the transcriptome [107]. HnRNPA2/B1 mediated m^6^A-dependent nuclear RNA processing in vivo and in vitro by associating with a subset of primary microRNA (miRNA) transcripts through m^6^A binding. This promoted miRNA processing by recruiting the microprocessor complex Drosha and DGCR8 [107]. Additional studies showed that hnRNPA2/B1 enhanced the stability of interleukin-binding factor 3 (ILF3) in an m^6^A-dependent manner to promote proliferation in multiple myeloma cells [108]. However, other investigators proposed that hnRNPA2/B1 mediated the effects of m^6^A through a switch mechanism, in which hnRNPA2/B1 may promote accessibility to specific binding sites, inducing primary miRNA processing, rather than functioning as a reader [109]. Further studies are warranted to unveil the role of hnRNP in regulating m^6^A-mediated effects in normal and diseased conditions.

### 3.5. mRNA Stability

Maintaining mRNA stability is an important mechanism to regulate gene expression, wherein certain proteins bind to specific mRNA *cis*-acting sequences. The length and sequences of the 3′UTR regions are determinants of mRNA half-life [110]. HnRNP affect the stability of various mRNAs by binding to specific sequences located in 3′UTRs. Several studies mapped the direct interactions of hnRNP with RNA using CLIP analyses [45,94,111]. These studies identified the AU-rich element (ARE), which consists of AUUUAA repeats mediating the degradation of mRNAs [112]. HnRNPK binds ARE elements in the interferon-γ-inducible protein (IP-10) 3′UTR, increasing IP-10 mRNA stability and thus its accumulation in monocytes, as demonstrated using RNA-affinity capture and luciferase reporter assays. Interestingly, methylation of hnRNPK increased the stability of IP-10 [113], suggesting additional mechanisms involved in fine-tuning this regulation. HnRNPA2/B1 functions as a trans-acting element to bind to particular motifs, called structural RNA stability motif 1 (sRSM1), located in the 3′UTR, to increase transcript stability, as demonstrated by RNA-immunoprecipitation-coupled sequencing (RIP-chip) and HITS-CLIP. HnRNPA2/B1 suppression decreased mRNA expression levels carrying sRSM1 [114]. The binding of hnRNPA2/B1 to U16 elements in the 3′UTR of C-P4H-α(I) and glucose transporter 1 (GLUT1) mRNA promotes mRNA stability, as shown in human fibrosarcoma and brain tumors, respectively [115,116]. Additionally, hnRNPA2/B1 also impacts the selection of polyadenylation sites in pre-mRNAs, thereby affecting mRNA half-life. The deletion of hnRNPA2/B1 leads to alternative polyadenylation site selection and regulation of 3′UTR lengths in several transcripts, as revealed by RNA-seq analyses of mouse spinal cord samples [117].

Other studies have shown that hnRNP also participate in mRNA degradation. Lack of hnRNPK significantly increased the mRNA stability of KLF4 and ZNF750, two molecules involved in the regulation of cell differentiation [118]. Similarly, hnRNPA2/B1 and hnRNPA1 bind to UAASUUAU sequences (UAACUUAU and UAAGUUAU), found in the 3′UTRs of ~1315 human mRNAs to induce mRNA degradation. Their association helps recruit the CCR4-NOT deadenylase complex, and induces the deadenylation of mRNA, thereby promoting mRNA decay [119]. HnRNPA1 regulates the NF-κB pathway by diminishing the stability of the inhibitor of apoptosis cIAP1 mRNA. Silencing of hnRNPA1 restored the mRNA levels of cIAP1 [120]. Altogether, these studies demonstrate that hnRNP play a significant role in regulating the mRNA stability of cancer-related genes, thereby promoting tumor progression.

## 4. Role of hnRNP in Cancer

**Figure 3:**
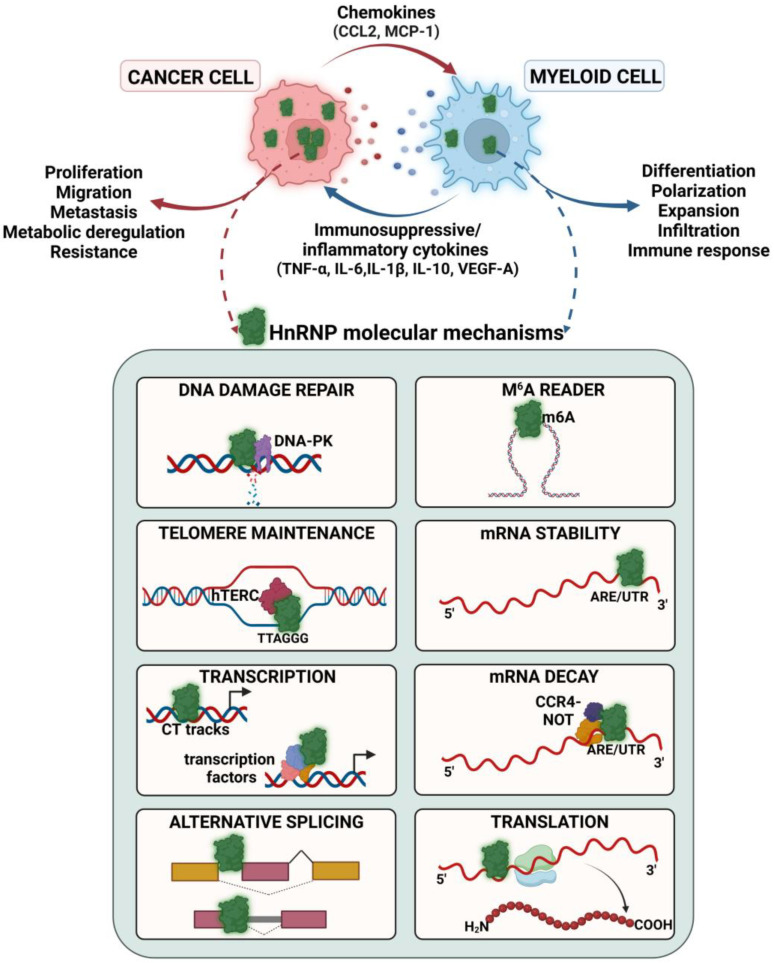
Various hnRNP-mediated molecular mechanisms regulate cancer-immune crosstalk. HnRNP increase tumor proliferation, migration, metastasis, metabolic deregulation, and therapeutic resistance. In myeloid cells, hnRNP can induce cell differentiation, polarization, expansion, infiltration, or act either as activators or repressors of the immune response. HnRNP in cancer cells regulate the production of chemokines that recruit myeloid cells into the TME. On the other hand, in myeloid cells, hnRNP control the generation of immunosuppressive or inflammatory cytokines to fuel uncontrolled tumor growth and metastasis. HnRNP-mediated effects in cancer and myeloid cells create a crosstalk, promoting a pro-tumor landscape.

Cancer is associated with proteomic alterations, playing major roles in uncontrolled tumor growth, metabolic rewiring, metastasis, and resistance to apoptosis [3]. The functions of hnRNP discussed in detail in the previous sections, including AS, regulation of mRNA stability, and transcription, are key in modulating the cancer proteome repertoire (Figure 3). High expression of hnRNP is characteristic of several cancer types, and their upregulation has been associated with poor patient survival, making them promising biomarkers for diagnosis [13]. For example, elevated expression of hnRNPK in tumors from gastric cancer patients correlated with poor survival [121]. Upregulation of hnRNPA2/B1 is associated with a lower overall and relapse-free survival rate in breast cancer patients, as shown by Kaplan–Meier survival analysis [10,122]. Immunohistochemistry detected a positive expression of hnRNPA2/B1 in 56.5% of primary invasive breast cancers and 9.7% of normal breast tissues, thus supporting the use of hnRNPA2/B1 as a clinical breast cancer diagnostic biomarker [123]. Similarly, hnRNPA2/B1 was identified as a potential biomarker for the early detection of lung cancer [124].

HnRNP-mediated control of tumor progression affects several signaling pathways critical in cancer. For instance, the downregulation of hnRNPA2/B1 suppressed the activation of the STAT3 pathway, inhibiting proliferation and in vivo tumorigenicity of breast cancer cells [125,126]. Additionally, hnRNPA2/B1 plays an important role in the survival of KRAS-dependent pancreatic cancer cells, wherein it directly interacts with and regulates the activity of mutated KRAS G12V and G12D to promote the PI3K/AKT/mTOR signaling pathway [127]. HnRNPH1 increased the growth and proliferation of chronic myeloid leukemia cells via the Akt pathway [128]. The phosphorylation of hnRNPK at serine 379 increased the expression of migratory molecules β-catenin and matrix metalloproteinase MMP12, thereby enhancing metastasis in the breast cancer cell line MDA-MB-231 [129]. However, a few studies have reported conflicting anti-tumorigenic effects of hnRNP in cancer. For example, hnRNPK haploinsufficiency reduced survival and enhanced hematopoietic neoplasms in mice by increasing p21 and STAT3 activation [130]. Overexpression of hnRNPK inhibited gastric tumor growth in vivo by activating the tumor-suppressing p53 pathway [131]. HnRNPA2/B1 reduced breast cancer cell proliferation and migration in vitro and in vivo, an observation contradictory to other reported studies [10,125] (Figure 3).

HnRNP can promote tumor progression by functioning as a transcriptional co-activator. HnRNPA2, acetylated by transcriptional co-activator p300, increased the expression of COX-2 and promoted the proliferation of non-small lung cancer cells [79]. HnRNPA1 recognizes the specific G4-DNA conformation of KRAS and forms an EGF-KRAS-ILK-hnRNPA1 regulatory loop to maintain the invasive activity of pancreatic PDAC cells [83]. HnRNPK interacts with and stabilizes yes-associated protein (YAP) on target gene promoters in TNF-α-stimulated hepatic progenitor cells to promote tumor development [132].

Related to its role as an m^6^A reader, toll-like receptor (TLR)-4 m^6^A sites were enriched with hnRNPA2/B1, as shown using genome-wide and methylation sequencing studies, thereby increasing proliferation in multiple myeloma cells [133]. Similarly, hnRNPA2/B1 indirectly enhanced the expression of Akt3 by increasing the stability of ILF3 through m^6^A site recognition to promote proliferation in multiple myeloma cells [108].

HnRNP induce tumor migration and metastasis through AS (Figure 3). HnRNPA1 enhanced lung cancer metastasis by modulating the splicing of *LAS1L* exon 9 [134]. HnRNPA2/B1-induced AS of *TP53INP2* inhibited migration in ovarian cancer cell lines [101]. HnRNPA2/B1 also promoted AS through exon skipping in apoptotic enzyme caspase-9 (*CASP9*) and oncogene *Ron* to generate their respective cancer-promoting isoforms in glioblastoma cells [135]. Regulation of hnRNP-driven splicing events can be potential targets of anti-cancer agents. For instance, we showed that the flavone apigenin, which associates directly with hnRNPA2, reverted the splicing of *c-FLIP* and *CASP9* to their respective pro-apoptotic variants found in non-carcinogenic cells [136]. Similarly, apigenin sensitized lung cancer cells to tumor necrosis factor-related apoptosis-inducing ligand (TRAIL) by increasing the protein levels of the TRAIL receptor (DR5) and reducing the expression of c-FLIP through AS rewiring. This effect enhanced TRAIL-induced apoptosis in human primary lung cancer epithelial cells [137]. Apigenin also sensitized breast cancer cells to the chemotherapeutic agent doxorubicin in an hnRNPA2-dependent manner [10]. These results support the ability of dietary compounds to modulate cancer through changes in hnRNP-mediated AS.

HnRNP can also promote cancer by binding and stabilizing lncRNAs. *CASC11* binds to hnRNPK and promotes the WNT/β-catenin pathway to induce tumor growth and metastasis in colorectal cancer [138]. eCLIP studies showed that HnRNPA2 binds to lncRNA *HOTAIR* and promotes breast cancer [45]. The lncRNA *EGOT* recruits hnRNPH1 to enhance the AS of pre-inositol 1,4,5-trisphosphate receptor type 1 (ITPR1) to promote autophagy in human breast cancer [139].

HnRNP also increase anti-cancer drug resistance in tumors (Figure 3). This effect is mediated by their effect on AS or mRNA stability. For example, hnRNPA2/B1 alters Bcl-x AS, increasing the Bcl-xL (long) anti-apoptotic splicing isoform, which induces breast cancer cell proliferation and reduces sensitivity to doxorubicin [140]. HnRNPA2/B1 knockdown reduced the resistance of breast cancer cell lines to the chemotherapeutic drug tamoxifen by diminishing the expression of estrogen receptor ERα and phosphorylated Akt, thereby leading to cell death [141]. HnRNPA2/B1 also reduced the sensitivity of breast cancer cells to olaparib by interacting with m^6^A sites in the 3′UTR of autophagy gene *ATG4B* to induce degradation [142]. Similarly, hnRNPK enhanced the expression of autophagy protein LC3I/II and increased the resistance to doxorubicin in acute myeloid leukemia [143]. Knockdown of hnRNPA1 re-sensitized enzalutamide-resistant prostate cancer cells by inhibiting the splicing of androgen receptors (AR) and thereby reducing the expression of AR-V7 [144]. High expression of hnRNPD in human pancreatic cells is correlated with resistance to gemcitabine [145]. Together, these studies highlight the role of hnRNP as cancer drivers, proposing that targeting hnRNP can provide opportunities to prevent cancer or improve the efficacy of commonly used anti-cancer treatments.

## 5. Role of hnRNP in Myeloid Cell Immune Regulatory Activity

Myeloid cells, including, among others, monocytes, macrophages, and MDSC, play a vital role in the TME, promoting cancer growth and resistance [4,5]. Myeloid cells are key drivers of inflammation. Cancer-related inflammation is recognized as an emerging cancer hallmark [3]. Increased macrophage recruitment to tumors is associated with enhanced metastasis of various cancer types [146]. Tumor-associated macrophages (TAM) promote a cancer-fueling transcriptional landscape and are associated with poor patient outcomes [147]. TAM also stimulate an immunosuppressive environment which prevents T cells and natural killer cells from halting tumor cells during tumor progression [148]. While the role of hnRNP in cancer-related immune dysregulation is not known, a few studies on the role of hnRNP in immune cells are emerging.

Some studies have described the role of hnRNP in regulating myeloid cell fate (Figure 3). For example, hnRNPA0 expression levels decide the cell fate of hematopoietic stem cells. HnRNPA0 haploinsufficiency reprograms the differentiation of hematopoietic progenitors from monocytic to pro-granulocytic cells. HnRNPA0 knockdown reduced the expression of transcripts rich in AU elements, which are enriched in genes encoding cell growth and differentiation proteins such as EMR, IL-1R2, and CSF1R, as shown using microarray gene expression analyses [149]. HnRNPK also regulates myeloid cell differentiation and expansion. This effect is due to HnRNPK-mediated AS regulation of *RUNX1*, a transcription factor important for myeloid cell differentiation and proliferation [9]. HnRNPE2 suppressed myeloid differentiation by functioning as a splicing silencer in the MM6 myeloid cell line. This is mediated by hnRNPE2 binding to an intron in the 5′UTR of S100A9, a key molecule in monocyte differentiation and oxidative response, to regulate AS [150].

HnRNP regulate the immune response by affecting the expression of several inflammatory cytokines, which contribute to tumor-promoting inflammation (Figure 3). For example, hnRNPL associates with lncRNA *THRIL* in the *TNF-α* promoter region, increasing the expression of TNF-α in monocytic THP1 cells stimulated with the TLR2 ligand Pam3CSK4 [151]. HnRNPU increases the expression of *IL-8* and *IL-12p40* by associating with ARE elements to regulate mRNA stability in a lncRNA *FIRRE*-dependent manner in LPS-stimulated macrophages [152]. HnRNPA2/B1 also regulates cytokines. Synovial macrophages from hnRNPA2/B1 knockout mice showed reduced IL-23 and TNF-α levels, resulting in decreased arthritis-induced inflammation [20]. Additional evidence of the role of hnRNPA2/B1 in immune regulation is provided by its ability to bind viral DNA and proteins, enhancing the immune response in virally infected macrophages through increased production of interferon (IFN)-β [153,154]. Additionally, hnRNPA2/B1 interacts with lncRNA *lincRNA-Cox2*, promoting inflammation by enhancing the transcription of NF-κB-regulated pro-inflammatory genes in LPS-stimulated macrophages [155]. HnRNPC, hnRNPK, and hnRNPU induced the expression of the inflammatory nitric oxide synthase 2 (Nos2), an enzyme involved in tumor metastasis through the production of reactive oxygen species, in a bacteria-infected RAW264.7 mouse macrophage cell line [156]. HnRNP can also induce immunosuppressive cytokines such as IL-10, which reduces anti-cancer immune competence and immunotherapeutic efficacy. For instance, hnRNPD enhanced IL-10 mRNA and protein levels by binding to its 3′UTR in the LPS-treated human monocytic leukemia THP-1 cell line [157]. Similarly, hnRNPA1 increased transcription, promoting the expression of LPS-induced IL-10, as shown using luciferase reporter assays and siRNA-mediated knockdown studies in RAW264.7 macrophages and human primary monocytes [158].

In some cases, hnRNP can function as immune repressors. For instance, the binding of hnRNPM to *IL-6* pre-mRNA acts as a safeguard for the inflammatory response. HnRNPM inhibits the maturation of *IL-6* by preventing its splicing through intron 3 retention. Phosphorylation of hnRNPM triggers its release from *IL-6* mRNA, promoting IL-6 splicing and subsequent activation of LPS-stimulated macrophages [21]. Similarly, hnRNPUL1 repressed the expression of IL-6 and IL-1β in LPS-stimulated macrophages and in vivo mice by competing with NF-κB on binding κB sites at the promoter during an inflammatory response, as shown using DNA pull-down and ChIP assays [159]

HnRNP function as regulators to several key signaling cascades driving immune responses that are important in cancer biology (Figure 3). HnRNPA1, through its RRM domain, associates with the inhibitor of NF-κB (IκBα), as demonstrated using purified proteins in vitro and mouse erythroleukemia cell lines. This association leads to IκBα degradation, thereby increasing NF-κB transcriptional activity [160]. On the other hand, hnRNPK functions as an NF-κB repressor. HnRNPK decreased the translation of TAK1, a kinase essential for NF-κB activation, resulting in decreased *TNF-α* and *IL-1β* mRNA steady-state levels in unstimulated macrophages. This effect is mediated by the association of HnRNPK with U/CCC_(n)_ elements in *TAK1* 3′UTR. Upon LPS stimulation, hnRNPK dissociates from the mRNA, allowing TAK1 translation [161,162]. The NLRP3 inflammasome pathway, a driver of cancer and chronic inflammation, is also regulated by hnRNP [163]. Knockdown of hnRNPK suppressed the activation of the NLRP3 inflammasome, resulting in decreased inflammatory cytokines IL-1β and IL-8 expression in RAW264.7 macrophages [164].

Together, these studies show that hnRNP affect macrophage immune function by regulating gene expression, AS, and protein translation, which contribute to their tumor-promoting functions. Overall, these studies revealed our limited understanding of hnRNP in immune cell function. So far, studies on the role of hnRNP in other myeloid cells, such as myeloid-derived suppressor cells, are lacking. Future findings in this area will be impactful in understanding the effect of hnRNP in regulating immune competency in cancer and other chronic inflammatory diseases.

## 6. HnRNP: Modulators of Cancer-Immune Crosstalk

While there has been tremendous progress in understanding the role of hnRNP in regulating cancer cell behavior, their function in controlling the cancer-immune landscape remains poorly understood. The contribution of hnRNP to the cancer-immune landscape occurs at different levels. HnRNP can modulate the production of cytokines and chemokines in both tumor cells and macrophages, which are critical players in orchestrating the cancer-macrophage crosstalk in the TME (Figure 3) [165]. For example, hnRNPK and hnRNPM regulate TNF-α and IL-6 expression by controlling their transcription and AS, respectively [21,132]. HnRNPA0 binds to *TNFα*, *IL-6*, *COX-2*, and macrophage inflammatory protein-2 (*MIP-2*) mRNA to enhance their stability in stimulated macrophages [23]. Chemokines derived from tumor cells are fundamental for the recruitment of myeloid cells into the TME [166]. Lack of hnRNPU downregulated the expression of CC motif ligand (CCL)-2, a chemokine required for macrophage infiltration into the TME [167]. Vascular endothelial growth factor (VEGF)-A is another key chemokine involved in myeloid cell migration and angiogenesis. HnRNPL increases VEGF-A mRNA stability, elevating its expression in hypoxic human monocytes [168]. HnRNPA2/B1 is one of the many m^6^A readers upregulated in macrophages and promotes infiltration in gastroesophageal adenocarcinoma patients [169]. Tumor cells mediate the polarization of macrophages into an immunosuppressive M2 phenotype, which plays an essential part in cancer progression [146]. A recent study has suggested a potential role of hnRNP in macrophage polarization.

LncRNA *MRF* enhanced the expression of monocyte chemotactic protein (MCP)-1 in an hnRNPD-dependent manner in mesenchymal stem cells, thereby inducing monocyte recruitment and macrophage polarization, as shown using in vitro co-culture experiments [170]. Overall, these studies suggest a role of hnRNP as a regulator of cancer-immune crosstalk by controlling the production of cytokines and chemokines in cancer and myeloid cells that contribute to the pro-tumor environment, myeloid recruitment, and polarization (Figure 3).

Recent findings also suggest a favorable role of hnRNP as a target for immunotherapy [171]. A recent study using methotrexate packaged in tumor-derived particles switched the macrophage phenotype from immunosuppressive M2 to proinflammatory M1 by activating lysosomal P450 monooxygenases and IFN-β production through hnRNPA2/B1 [172]. HnRNPL knockdown reduced the expression of programmed death ligand (PDL)-1 in prostate cancer, which sensitized cancer cells to T cell infiltration and anti-PD-1 therapy [173]. TAM also play a key role in regulating immunosuppression through PD-1/PDL-1 [174,175]. These findings suggest the need to investigate the role of hnRNP in modulating PDL-1 in macrophages to enhance the efficacy of anti-PDL-1 immunotherapy. While these findings provide initial evidence of hnRNP as modulators of the cancer-immune landscape, the molecular mechanisms responsible for the role of hnRNP in cancer-immune crosstalk remain largely unexplored. Additionally, studies demonstrating how the function of hnRNP vary in different myeloid cell populations and the mechanisms controlling their immunosuppressive and inflammatory nature are warranted to gain a thorough understanding of their role in the TME.

## 7. Conclusions

The crosstalk between cancer and myeloid cells in the TME determines tumor initiation, progression, metastasis, and response to therapy. Over the last decades, significant evidence has demonstrated the role of hnRNP as cancer drivers, promoting tumor growth and metastasis by regulating major signaling pathways through AS, mRNA stability, or functioning as transcriptional modulators. Additional roles of hnRNP as readers recently emerged and will require further investigation. Studies in immune cells are beginning to reveal the role of hnRNP in immune regulation. Significant unmet goals, including understanding how hnRNP expression is regulated, and identifying specific targets and their function in non-cancer cells, warrant additional studies. Technical advances, including RNA-seq, ChIP, and CLIP-seq analyses, have helped reveal the mechanisms responsible for the role of hnRNP in cancer. The same approaches applied systematically in immune cells will uncover their role in immune regulation. Together, these studies will provide a better understanding of the function of hnRNP in the cancer-immune landscape, which should help improve the outcomes for the prevention and treatment of cancer.

## Data Availability

Not applicable.
